# Effect of Task Failure on Intermuscular Coherence Measures in Synergistic Muscles

**DOI:** 10.1155/2018/4759232

**Published:** 2018-06-03

**Authors:** Anna Margherita Castronovo, Cristiano De Marchis, Maurizio Schmid, Silvia Conforto, Giacomo Severini

**Affiliations:** ^1^Department of Bioengineering, Imperial College London, London SW7 2AZ, UK; ^2^School of Electrical & Electronic Engineering, University College Dublin, Belfield, Dublin 4, Ireland; ^3^Department of Engineering, University of Roma Tre, Via Vito Volterra 62, Rome, Italy

## Abstract

The term “task failure” describes the point when a person is not able to maintain the level of force required by a task. As task failure approaches, the corticospinal command to the muscles increases to maintain the required level of force in the face of a decreased mechanical efficacy. Nevertheless, most motor tasks require the synergistic recruitment of several muscles. How this recruitment is affected by approaching task failure is still not clear. The increase in the corticospinal drive could be due to an increase in synergistic recruitment or to overlapping commands sent to the muscles individually. Herein, we investigated these possibilities by combining intermuscular coherence and synergy analysis on signals recorded from three muscles of the quadriceps during dynamic leg extension tasks. We employed muscle synergy analysis to investigate changes in the coactivation of the muscles. Three different measures of coherence were used. Pooled coherence was used to estimate the command synchronous to all three muscles, pairwise coherence the command shared across muscle pairs and residual coherence the command peculiar to each couple of muscles. Our analysis highlights an overall decrease in synergistic command at task failure and an intensification of the contribution of the nonsynergistic shared command.

## 1. Introduction

Task failure is defined as the point at which a subject is not able to maintain the level of force needed to execute a task [1]. This mechanical outcome is the result of complex central and peripheral mechanisms governing the coordination of many muscles involved in the task execution.

It has been shown that during submaximal or maximal contractions sustained until voluntary exhaustion an increase in muscular activation occurs [2, 3] due to the progressive recruitment of muscle fibres [4]. At a more detailed level, task failure has been associated with an initial increase, followed by a decline, of the discharge frequency of the motor neuron pool [5, 6] and with an intensification of the neural drive to muscles [7] and of the high-frequency oscillations at the corticospinal level [8].

Albeit changes in the overall multimuscle coordination strategies have been associated with the occurrence of task failure in several studies [9–12], the way the central nervous system (CNS) coordinates the neural commands to multiple muscles in the presence of voluntary exhaustion is yet to be fully clarified.

It has been hypothesized that, in normal conditions, the regulation of movement by the CNS passes through the selective recruitment of low-dimensional spatiotemporal structures of muscle coactivation aiming at resolving the neuromusculoskeletal redundancy [13]. These motor modules, usually referred to as “muscle synergies,” are thus able to represent muscle coordination in a compact way during the execution of various movements under different biomechanical and physiological constraints [13, 14].

While the analysis of motor modules unravels the temporal coordination of motor commands across different muscles, it does not disclose the actual nature of the communality of the control signal activating each muscle. In the past, this information has been accessed using measures of spectral synchronicity, such as coherence [15], between the different signals associated to the execution of a motor task. Specifically, the frequency content of the neural command to muscles has been studied through the analysis of the coherence between motor unit spike trains within the same muscle [7, 16, 17] or between muscles [18]. Other approaches have investigated the coherence between cortical signals and peripheral muscle activation (corticomuscular coherence (CMC)) [19, 20] and between bipolar EMG signals coming from different muscles (EMG-EMG coherence or intermuscular coherence (IMC)) [10, 21].

In particular, IMC aims at investigating these neural mechanisms from a purely peripheral information [20, 22] focusing on the contributions within different frequency bands [23–25]. In fact, EMG-EMG coherence may reveal the presence of shared neural presynaptic input from higher brain structures and particularly from the motor cortex [26–28], but also common contributions between the spinal interneurons [20].

Previous studies on walking, cycling, manual dexterity, and upright posture maintenance have reported the presence of significant IMC at beta (15–30 Hz) and gamma (30–100 Hz) bands between pairs of synergistic muscles [24, 25, 29–31]. These studies suggest that the degree of correlation observed between the activity of different muscles can reflect the functional coactivation, and it might be extended to muscles that are either anatomically close and functionally similar [10, 25, 32] or anatomically distant and functionally different [29]. A recent study using coherence analysis on motor unit spike trains has reported that functionally coupled and anatomically close synergistic muscles share most of their synaptic inputs [18].

Thus, by combining the information obtained from the analysis of the modularity and spectral synchronicity of the motor command, it is likely possible to unravel deeper insights on the neuromuscular mechanisms of task failure. In this study, we investigated this possibility by studying the temporal and spectral correlates of the coordination of three synergistic muscles of the quadriceps femoris during dynamic knee extension tasks repeated at two different force levels until task failure. To quantify muscle coordination strategies and the communality in the neural drive to muscles, we integrated muscle synergy analysis with different measures of intermuscular coherence.

To highlight the coherence contributions that are common to all synergist muscles or unique to each pair of muscles, we measured both the pooled coherence [33] across the three muscles and the pairwise coherence [34] among each pair of muscles. To isolate components of coherence that are uniquely associated with each muscle pair, we also estimated the residual pairwise coherence [18, 35] that represents the coherence between two signals after the removal of the components that are synchronized with a third one. In this study, we estimated the residual coherence of each pair of muscle after removal of the components that are common to the third muscle that in this case can be accounted as the synergistic coherence contribution. In this way, we tested whether task failure alters the different measures of coherence in different ways, thus providing a deeper insight onto the synergistic muscle recruitment mechanisms at task failure.

## 2. Materials and Methods

### 2.1. Participants

Eleven healthy individuals (27 ± 5 years of age, 3 females) participated in this study. Inclusion criteria consisted in the absence of any neurological, orthopedic, or cognitive impairment that would in any way affect the execution of the experiment. All procedures and data collections were carried on at the Biolab^3^ of Roma Tre University. All participants agreed to participate to the study by signing an informed consent. All procedures were conducted in accordance with the policies of the Applied Electronics section of the Department of Engineering at Roma Tre University and with the Declaration of Helsinki.

### 2.2. Experimental Procedures

**Figure 1:**
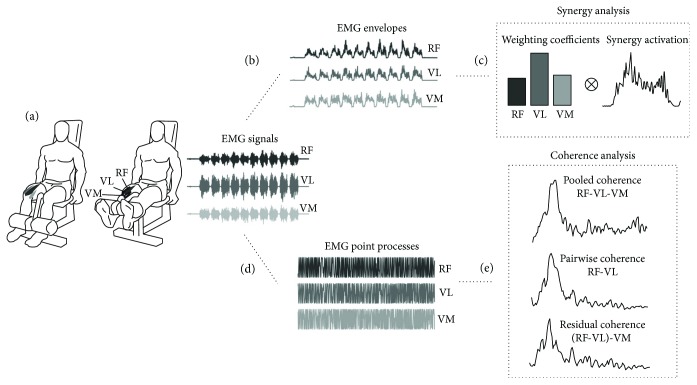
Experimental setup and data analysis. (a) Experimental setup. Subjects were seated on a leg extension machine in an upright position. They were asked to perform repetitions of a knee extension task until task failure. Surface EMG signals were recorded from three muscles of the quadriceps: rectus femoris (RF), vastus lateralis (VL), and vastus medialis (VM). (b) EMG signals were band-pass filtered between 30 and 450 Hz, full-wave rectified, and low-pass filtered with a cutoff frequency of 15 Hz to extract the envelope. (c) Nonnegative matrix factorization algorithm was applied to data for LIE and HIE to reconstruct the activity of the three muscles as a single motor module *W* containing the relative activation weights of the three muscles as recruited by an activation signal *H* so that *M_r_ ≈ WxH* where *M_r_* is the reconstructed matrix. (d) For the coherence analysis, EMG signals were detrended demodulated by means of Hilbert transform. (e) Then, coherence analysis was performed across the three muscles together (pooled coherence), between pairs of muscles (pairwise coherence) and between pairs of muscles after removing components common to the third muscle (residual coherence).

All subjects underwent two testing sessions in two different nonconsecutive days. At the beginning of each testing session, subjects were asked to sit on a leg extension device (leg extension ROM, Technogym). Subjects were strapped to the leg extension device to maintain a 90-degrees hip angle and to avoid possible compensations with the trunk during the different exercises. Subjects were asked to conduct a preliminary test to determine the maximum amount of weight they could lift and hold with both legs for 5 s during a knee extension exercise. Subjects then performed a series of repetitions of the knee extension at either 20% (low-intensity exercise (LIE)) or 70% (high-intensity exercise (HIE)) of their maximal lifting weight (Figure 1(a)).

The order of exercise (LIE or HIE) was randomized between the two testing days across subjects. Each exercise consisted of consecutive series of 10 dynamic contractions separated by 5 s of rest. Subjects were instructed to perform the contractions as fast as they could without stopping until task failure, which was defined as the first series that each participant was not able to complete (e.g., the participant was not able to perform the full range of motion of the movements). Surface EMG signals were recorded from the rectus femoris (RF), vastus medialis (VM), and vastus lateralis (VL) of the dominant leg (defined as the leg the subjects would use to kick a ball) of each subject during each exercise (Figure 1(a)). EMG signals were recorded using a wireless system (BTS FREEEMG, http://btsbioengineering.com), sampled at 1000 samples/s and digitized at 14 bits.

### 2.3. Amplitude and Motor Module Analysis

We analyzed the coactivation of the three muscles during the different exercises using factorization analysis. Initially, the EMG signals of the three muscles were band-pass filtered between 30 and 450 Hz, full-wave rectified, and low-pass filtered with a cutoff frequency of 15 Hz to extract the envelope (Figure 1(b)). For each subject and each exercise (LIE and HIE), we extracted the first series of 10 dynamic movements as representative of the *baseline* condition of the experiment and the last series of 10 dynamic movements as representative of the *task failure* condition. Changes in amplitude of the three EMGs between *baseline* and *task failure* were estimated by calculating the root mean square (RMS) of the signals. We applied the nonnegative matrix factorization (NMF) algorithm [36] to the data of each condition to reconstruct the muscular activity of the three muscles (matrix *M*) as a single motor module *W* containing the relative activation weights of the three muscles as recruited by an activation signal *H* so that *M_r_ ≈ WxH* where *M_r_* is the reconstructed matrix (Figure 1(c)). The quality of the reconstruction was determined by calculating the *R*^2^ value between the original matrix *M* and *M_r_*. For each analysis of each subject, *W* was normalized by its norm, to allow for comparison across different recordings. Changes in amplitude of the activation pattern of the motor module *W* between the different conditions were estimated by calculating the RMS of *H*.

### 2.4. Pooled, Pairwise, and Residual Coherence Analysis

Coherence analysis was used to assess the linear dependency between the spectral components of the different muscles during each task. Coherence was studied across the three muscles together (pooled coherence [33]), between couples of muscles (pairwise coherence [15]) and between couples of muscles after removing components common to the third muscle (residual coherence [18]) (Figure 1(e)). The same preprocessing was applied to the EMG signals prior to each coherence analysis. EMG signals were detrended, but no band-pass filtering was applied to the signals to avoid effect of filtering in the coherence analysis (Figure 1(d)). In agreement with our previous work, and in order to limit possible detrimental effects of the rectification process [37], coherence analysis was performed on the demodulated point process of the dynamic nonrectified EMG signals by removing the slow-varying amplitude modulation of the signals [25]. This was achieved by means of a demodulation procedure based on Hilbert transform [21]. This step was necessary as the amplitude modulation of the dynamic knee extension contractions constitutes a limit to the stationarity requirements of coherence estimation. Specifically, the instantaneous frequency of the analytic signal for each EMG signal *x(t)* was estimated as

(1) $$\begin{array}{c} \theta \left(t\right)=\tan^{-1}\left[\frac{x_{H}\left(t\right)}{x\left(t\right)}\right], \end{array}$$

where *x_H_*(*t*) represents the Hilbert transform of the signal. As shown by Boonstra and colleagues [21], the demodulated EMG signal can then be obtained from the instantaneous frequency as

(2) $$\begin{array}{c} x_{D}\left(t\right)=\cos\left[\theta \left(t\right)\right]. \end{array}$$

The values of *x_D_*(*t*) span the range [−1,1]. To limit spurious coherence contributions due to measurement noise and common to the three channels in absence of signals (e.g., during the interburst of the fast-paced dynamic contractions), an activity detection algorithm [38] was applied to the raw EMG signals *x*(*t*) to estimate the portions of the signals when the three muscles were simultaneously active. For each EMG signal, a time series *x_DC_*(*t*) was constructed by concatenating the parts of the EMG signals where all three muscles were active. A Hanning window of the same length multiplied each coactivation segment, before concatenation, to avoid abrupt transitions. For each exercise (LIE and HIE) of each subject, the first 10 seconds of the *x_DC_*(*t*) signal was extracted as representative of the *baseline* condition, while the last 10 seconds was extracted as representative of the *task failure* one. All coherence analyses were then performed separately on the dataset represented by the *baseline* and *task failure* conditions of each subject during each experiment. The common neural coupling between the three muscles was estimated by means of the pooled coherence function [33], defined as follows:

(3) $$\begin{array}{c} C_{pool}\left(f\right)=\frac{{\left|\sum_{j=1}^{p}P_{xy}\left(f\right)L_{j}\right|}^{2}}{\left(\sum_{j=1}^{p}P_{{xx}_{j}}L_{j}\right)\left(\sum_{j=1}^{p}P_{{yy}_{j}}L_{j}\right)}, \end{array}$$

where *p* represents all the possible pairs of muscles (3 in our case, namely RF-VM, RF-VL, and VM-VL), *j* denotes the *j*th pair, *P_xy_*(*f*) is the power cross-spectral density, *P_xx_*(*f*) and *P_yy_*(*f*) are the autospectral densities of the two muscles forming the couple, and *L_j_* is the number of segments used for the cross-spectrum and autospectra estimation. *P_xy_*(*f*), *P_xx_*(*f*), and *P_yy_*(*f*) were estimated on segments lasting 500 ms (windowed using a Hanning function) with 50% overlap [39] leading to a spectral resolution of 2 Hz, while doubling the number of available segments to improve estimation. Pairwise coherence analysis was used to estimate the coherence contribution between two muscles. This analysis is based on the standard coherence formulation:

(4) $$\begin{array}{c} C_{xy}\left(f\right)=\frac{{\left|P_{xy}\left(f\right)\right|}^{2}}{\left|P_{xx}\left(f\right)\right|\left|P_{yy}\left(f\right)\right|}. \end{array}$$

Coherence was calculated from the *x_DC_*(*t*) time series of all possible pairs of muscles for the *baseline* and *task failure* conditions of each exercise. Also in this case, the autospectra and the cross-spectra were calculated using Welch's method on 500 ms portions windowed using Hanning function and with 50% overlap. Finally, we wanted to analyze coherence contributions between pairs of muscles after removal of the components that are synchronous with the activity of the third muscle, thus coherence contributions that were common only to those two muscles. The mathematical formulation of residual coherence was the same utilized by Laine and colleagues on motor unit spike trains [18]. Specifically, given 3 time series *x*(*t*), *y*(*t*), and *z*(*t*), whose autospectra are *P_xx_*(*f*), *P_yy_*(*f*), and *P_zz_*(*f*), respectively, and given all the possible combinations of cross-spectra between the three time series, the residual autospectra and cross-spectrum between *x*(*t*) and *y*(*t*) while excluding the components common to *z*(*t*) can be calculated as

(5) $$\begin{array}{c} P_{xx-z}\left(f\right)=P_{xx}\left(f\right)-\frac{P_{xz}\left(f\right)P_{zx}\left(f\right)}{P_{zz}\left(f\right)},\\ P_{yy-z}\left(f\right)=P_{yy}\left(f\right)-\frac{P_{yz}\left(f\right)P_{zy}\left(f\right)}{P_{zz}\left(f\right)},\\ P_{xy-z}\left(f\right)=P_{xy}\left(f\right)-\frac{P_{xz}\left(f\right)P_{zy}\left(f\right)}{P_{zz}\left(f\right)}. \end{array}$$

The residual coherence between *x*(*t*) and *y*(*t*) can then be calculated as

(6) $$\begin{array}{c} C_{xy-z}\left(f\right)=\frac{{\left|P_{xy-z}\left(f\right)\right|}^{2}}{\left|P_{xx-z}\left(f\right)\right|\left|P_{yy-z}\left(f\right)\right|}. \end{array}$$

The residual coherence *C_xy−z_* (*f*) was calculated for all three combinations of the time series *x_DC_*(*t*) associated with the three muscles. Also in this case autospectra and cross-spectra were calculated using 500 ms Hanning windows with 50% overlap. Each estimated coherence profile (pooled, pairwise, and residual) underwent Fischer transformation to normalize the coherence contributions and to allow for comparisons among different participants. The Fischer transformation was defined as follows:

(7) $$\begin{array}{c} Z_{xy}\left(f\right)=\sqrt{2L}\tanh^{-1}\left(\sqrt{C_{xy}}\right), \end{array}$$

where *L* is the number of windowed segments used for the estimation of the coherence profile. All the Fischer-transformed coherence spectra were then smoothed using a 3-point average filter. Coherence analysis is always paired with an estimation of the significance level for the derived coherence spectra. In this work, the confidence level was estimated by performing a surrogate data analysis approach. Surrogate series were generated for each EMG signal *x*(*t*) by calculating the Fourier transform, independently shuffling the phase components, and calculating the inverse Fourier transform [40, 41]. This procedure ensures the preservation of the original power spectrum in the surrogate series while making the original and surrogate series completely uncorrelated in the time domain and frequency domain. For each coherence spectrum, 50 surrogate sets of EMG signals were used to calculate a set of coherence spectra expected from chance. The significance level was then calculated as the 95% percentile of the surrogate coherence spectra.

### 2.5. Statistical Analysis

An initial statistical analysis was performed to assess for differences in the time to task failure between the LIE and HIE exercise intensities (Wilcoxon's signed rank test, *α* = 0.05). We performed a series of statistical tests to assess for differences between the *baseline* and *task failure* conditions for time and frequency domain features extracted in both the LIE and HIE exercises. In the time domain, we assessed if transition from *baseline* to *task failure* was characterized by changes in the amplitude of the EMG signals and in the shape and magnitude of activation of the single motor module extracted from the three muscles. Specifically, we tested for changes in the RMS of each specific muscle between *baseline* and *task failure*. We also tested for significant changes in the weights of each single muscle in the matrix *W* obtained from the (NMF) algorithm and for changes in the RMS of the vector *H* representing the time-dependent activation of the module *W*. All these analyses were based on Wilcoxon's signed rank test. For the coherence analyses, we tested for differences between *baseline* and *task failure* in the average significant gamma band (30–100 Hz) values of the pooled, pairwise, and residual coherence profiles extracted in both LIE and HIE. Moreover, we tested for significant changes in the contribution of each residual coherence spectrum in the corresponding pairwise coherence spectrum. This parameter (*C_perc_*) was calculated as the average percentage contribution of the residual coherence in the pairwise coherence, as follows:

(8) $$\begin{array}{c} C_{perc}=\frac{1}{f}\cdot \underset{f}{\sum }\frac{100\cdot C_{R}\left(f\right)}{C_{p}\left(f\right)}, \end{array}$$

where *C_P_* (*f*) represents the pairwise coherence and *C_R_* (*f*) represents the residual coherence. Also, in this case, all statistical analyses were based on Wilcoxon's signed rank test. To limit the possibility of type I errors that may incur due to the multiple comparisons across the different muscle pairs, the *P* values obtained from the tests were adjusted using Benjamini and Yekutieli procedure [42] for controlling the false discovery rate (FDR) with FDR level set at 0.05.

## 3. Results

As expected, time to task failure was reported to be significantly different between exercise intensities. Specifically, task failure occurred on average after 1231 ± 572 seconds for LIE and after 234 ± 71 seconds for HIE. This difference was shown to be statistically significant (*P* < 0.01).

### 3.1. Changes in EMG Amplitude and Shape and Activation of the Motor Module

Table 1 shows the results on the analysis of the RMS of the three muscles between the *baseline* and *task failure* conditions for both the LIE and HIE experiments. As expected, for all three muscles in both experiments, we found that *task failure* was associated with a statistically significant (*P* < 0.01 for all six comparisons) higher muscular activation as estimated using the RMS analysis.

**Figure 2:**
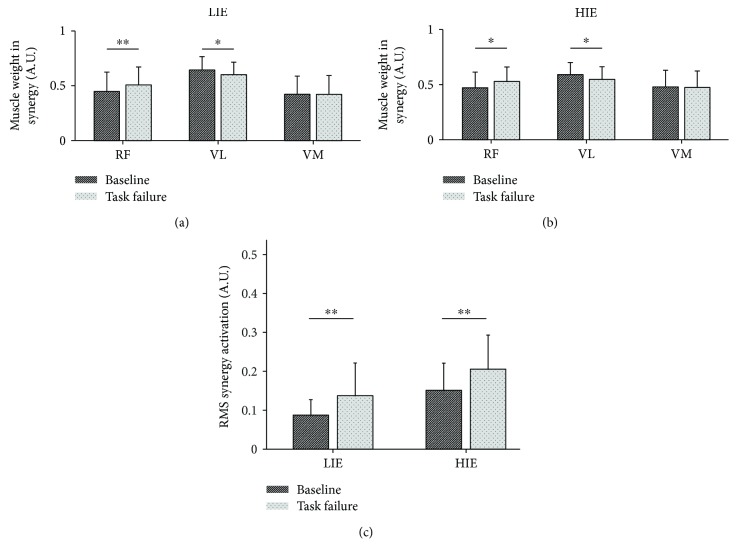
Changes in the muscle module at task failure. The synergistic activation of the RF, VL, and VM in the knee extension task is exploited using the muscle synergy framework. Muscle weighting coefficients are reported for each muscle and each condition (baseline versus task failure) for both (a) LIE and (b) HIE. (c) The compound synergy activation is reported for both LIE and HIE at baseline and task failure. Significance is reported for the comparison baseline versus task failure. ^∗^*P* < 0.05, ^∗∗^*P* < 0.01. RF: rectus femoris; VL: vastus lateralis; VM: vastus medialis; LIE: low-intensity exercise; HIE: high-intensity exercise. All bar plots are presented as the mean ± standard deviation.

As the three muscles under analysis activate together during knee extension movements, in our motor module analysis, we fixed the NMF decomposition to one single module. As confirmation of our choice, we found that the activity of the three muscle actively participating in the knee extension task could be well reconstructed as the activation of a single motor module. Specifically, the factorization based on the NMF algorithm yielded average (across subjects) *R*^2^ values between the original and reconstructed envelopes of 0.89 ± 0.06 and 0.88 ± 0.03 for the LIE experiment and correspondent values of 0.88 ± 0.06 and 0.89 ± 0.04 for the HIE experiment, for the *baseline* and *task failure* conditions, respectively. Moreover, the motor module shape showed remarkably consistent trends between the LIE and HIE conditions. In particular, a significant increase in the contribution of the RF (*P* < 0.01 for LIE and *P* = 0.014 for HIE) to the motor module, coupled with a contemporary decrease in the contribution of the VL (*P* = 0.02 for LIE and *P* = 0.02 for HIE), was observed passing from *baseline* to *task failure* (Figures 2(a) and 2(b)). Changes in the contribution of the VM muscle between *baseline* and *task failure* during both the LIE and HIE experiments were instead absent. Consistent with what was observed in the analysis of the RMS of the EMG signals, a significant increase in the RMS of the activation pattern of the motor module activation signals between the *baseline* and *task failure* conditions in both experiments (*P* < 0.01 for LIE and *P* < 0.01 for HIE) was noticed (Figure 2(c)).

### 3.2. Pooled Coherence Analysis

**Figure 3:**
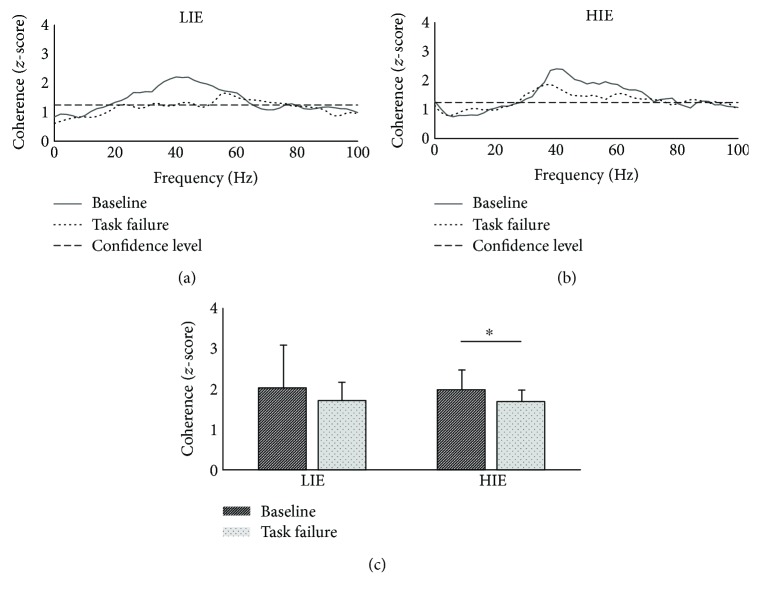
Changes in the pooled coherence at task failure. Pooled intermuscular coherence profiles are reported for (a) LIE and (b) HIE. Solid black line represents the baseline condition. Dashed line represents the task failure one. Dotted line is used to depict the confidence level. (c) Average maximum values of the pooled intermuscular coherence across all subjects for both LIE and HIE. Significance is reported for the comparison baseline versus task failure. ^∗^*P* < 0.05. LIE: low-intensity exercise; HIE: high-intensity exercise. All bar plots are presented as the mean ± standard deviation.

In agreement with the results we previously obtained during pedaling task [25], we observed values of pooled coherence above the significance level only in the 30–100 frequency band, usually referred to as gamma band. Figures 3(a) and 3(b) display the average (across subjects) pooled coherence spectra, expressed in *z*-scores, for the LIE and HIE experiments at *baseline* (solid lines) and *task failure* (dashed lines).

In both experiments, we reported a marked decrease in pooled coherence during *task failure*. This visual observation is partially confirmed by the statistical analysis on the average coherence *z*-scores observed in the gamma band. For the LIE experiment, we reported an across-subjects average *z*-score of 2.12 ± 1.10 at *baseline* which decreased to 1.84 ± 0.46 in the *task failure* condition. This observed change was found to be not significant (*P* = 0.42), possibly due to the high variability observed for the average coherence *z*-score at *baseline*. For the HIE experiment, we observed an across-subjects average *z*-score of 2.07 ± 0.51 at *baseline* and a decreased value of 1.76 ± 0.29 at *task failure* (*P* = 0.04).

### 3.3. Pairwise and Residual Coherence Analysis

**Figure 4:**
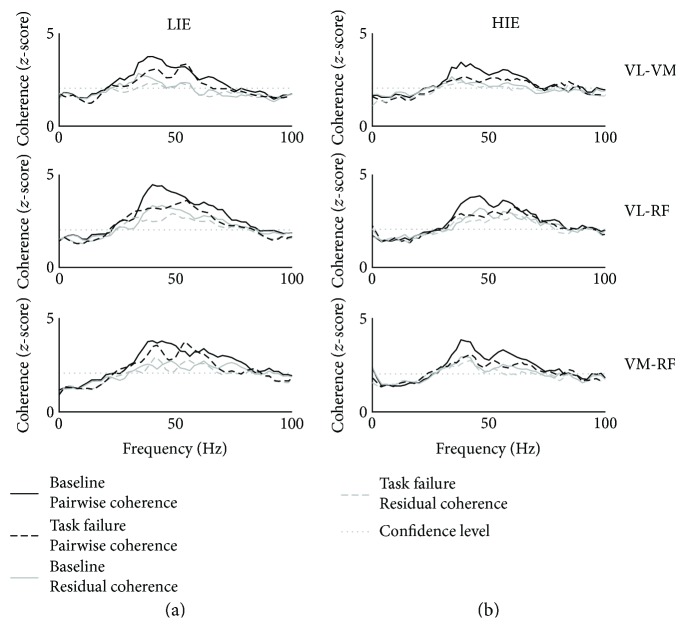
Pairwise coherence. Pairwise *z*-score intermuscular coherence profiles (solid lines) as averaged across all subjects for baseline (solid black line) and task failure (solid grey line) and residual pairwise *z*-score intermuscular coherence profiles (dashed lines) as averaged across all subjects for baseline (dashed black line) and task failure (dashed grey line). Profiles are depicted for the conditions (a) LIE and (b) HIE and for the pairs (*from the top*): VL-VM, VL-RF, and VM-RF. Dotted black line represents the confidence level. RF: rectus femoris; VL: vastus lateralis; VM: vastus medialis; LIE: low-intensity exercise; HIE: high-intensity exercise.

Pairwise coherence further confirmed the results obtained for pooled coherence. The panels (a and b) in Figure 4 show the average (across subjects) *z*-scores for the pairwise coherence profiles of all the muscle couples during the LIE and HIE tasks, respectively. For all three muscle pairs, coherence values were above confidence level only in the gamma range. Also, in this case, the *task failure* condition (dashed black line) was associated with lower values of coherence with respect to the *baseline* one (solid black line) in both the LIE and HIE experiments. Residual coherence plots (grey lines, solid for *baseline* and dashed for *task failure*) followed closely the respective pairwise profiles while showing lower coherence values. Different from what was observed for the pairwise profiles, we did not notice obvious differences in the magnitude of the residual coherence between the two conditions.

**Figure 5:**
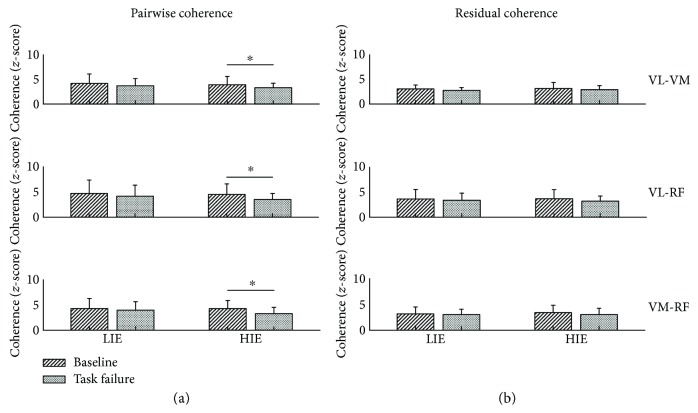
Changes in gamma pairwise and residual coherence at task failure. Average maximum value across all subjects in the range [30–100] Hz for the (a) pairwise intermuscular coherence and the (b) residual intermuscular coherence for the pairs (*from the top*): VL-VM, VL-RF, and VM-RF. Significance is reported for the comparison baseline versus task failure. ^∗^*P* < 0.05. RF: rectus femoris; VL: vastus lateralis; VM: vastus medialis; LIE: low-intensity exercise; HIE: high-intensity exercise. All bar plots are presented as the mean ± standard deviation.

Statistical analysis (Figure 5) showed significant changes in the average coherence *z*-score of each pair of muscles only for the HIE condition, while changes were not significant for the LIE condition. In the LIE experiment, a consistent decrease, although not significant, between *baseline* and *task failure* in the average significant coherence *z*-scores for all three muscle pairs was reported (*P* = 0.19 for VL-VM, *P* = 0.07 for VL-RF, and *P* = 0.35 for VM-RF). The same trend, although this time statistically significant, was observed for the HIE experiment (*P* = 0.01 for VL-VM, *P* = 0.01 for VL-RF, and *P* = 0.02 for VM-RF). For the residual coherence, we reported a slight decrease in the average significant gamma *z*-scores for both experiments, although these results were shown not to be statistically significant (LIE: *P* = 0.32 for VL-VM, *P* = 0.46 for VL-RF, and *P* = 0.43 for VM-RF and HIE: *P* = 0.90 for VL-VM, *P* = 0.32 for VL-RF, and *P* = 0.43 for VM-RF).

**Figure 6:**
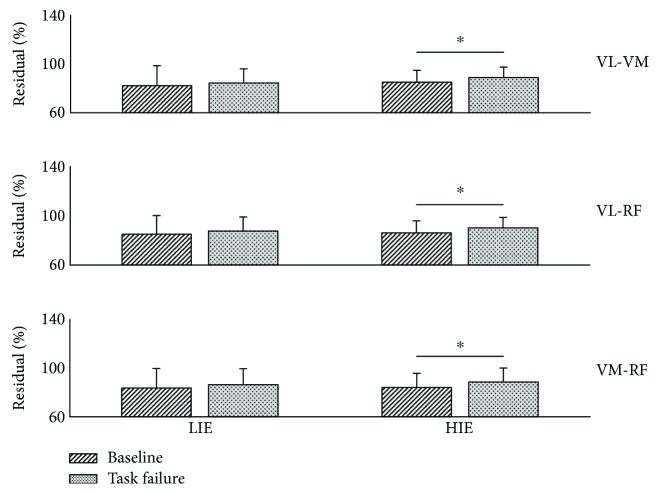
Percentage contribution of the residual coherence in the pairwise coherence. The subplots present the following muscle pairs (*from top to bottom*): VL-VM, VL-RF, and VM-RF. Significance is reported for the comparison baseline versus task failure. ^∗^*P* < 0.05. RF: rectus femoris; VL: vastus lateralis; VM: vastus medialis; LIE: low-intensity exercise; HIE: high-intensity exercise. All bar plots are presented as the mean ± standard deviation.

As final analysis, we evaluated the percentage contribution of the residual coherence to the pairwise coherence (see 8) for each muscle pair for both conditions and exercise intensity.  Figure 6 shows the results for this analysis. Once again, we observed significant changes only in the data extracted during the HIE exercise. For the VM-VL muscle pair, we found that, during the LIE exercise, the residual coherence accounted for 83.1 ± 17.7% of the total pairwise coherence in the *baseline* condition and 85.8 ± 12.8% for the *task failure* condition (*P* = 0.41). Similar results were observed also for the RF-VL and RF-VM muscle pairs (86.4 ± 16.3% versus 89.3 ± 12.6%, *P* = 0.24 for RF-VL, 84.6 ± 17.0% versus 87.6 ± 13.7%, *P* = 0.46 for RF-VM). In the HIE experiment, we again observed an increase in the relative contribution of the residual coherence in the pairwise coherence with values of 85.5 ± 11.0% at *baseline* and 90.8 ± 9.0% at *task failure* (*P* = 0.03) for VL-VM, 87.8 ± 10.5% and 92.2 ± 8.9% (*P* = 0.03) for RF-VL, and 85.1 ± 12.5% and 89.9 ± 12.0% (*P* = 0.03) for RF-VM.

## 4. Discussion

In this work, we investigated how task failure modifies both the synergy structure and the spectral synchronicity of three synergistic muscles of the quadriceps femoris during a knee extension task.

We found that, at task failure, the relative contribution of the three muscles to the synergy is modified. At the same time, we observed, only in the HIE task, a drop in pooled coherence that is echoed by a decrease in coherence between each muscle pair. Interestingly, we did not observe changes in the residual coherence spectra for each pair of muscles after the exclusion of the contributions synchronous with the activity of the third one. We interpret this latter measure as theoretically linked to a measure of coherence between two muscles after excluding the contributions relative to an underlying synergistic command common to all three muscles. In the following sections, we will expand upon the possible physiological mechanisms behind the observations made in our results.

### 4.1. Amplitude and Motor Module Analysis

The observed increase of EMG-RMS in all muscles is consistent with the previous studies in the literature showing an intensification of muscle activation concurrent with *task failure* during both static [43–46] and dynamic contractions [2, 47, 48]. This behaviour has been associated with a progressive recruitment of larger motor units in order to maintain the required level of force [49], even considering the limitations due to the amplitude cancellation in the generation of the EMG interference pattern [50].

The increase in the RMS profiles of the single muscles at *task failure* is reflected also in a significant intensification of the overall synergy activation for both exercise intensities (Figure 2). Previous studies on muscle coordination have suggested that the CNS has a tendency to change the activation level of the single muscle rather than to modify the motor modules structure which are then invariant to physiological and biomechanical constraints [12, 51, 52]. In our study, the observation that the synergy between RF, VL, and VM is robust at the *baseline* level between the two exercise intensities further supports this theory. However, this robustness seems not to be maintained at *task failure*.

The changes that we observed in the synergy weighting coefficients while approaching voluntary exhaustion in both LIE and HIE suggest, in fact, a modification in the strategy of coactivation of the three muscles for supporting the knee extension while approaching *task failure*. This hypothesis is further confirmed by the fact that the weighting coefficients vary in a consistent way across exercise intensities. A similar behaviour of alternate activity among synergistic muscles has already been observed in the past in the triceps surae and quadriceps muscles during a fatiguing task [53–55]. Due to both the analytical constraints imposed in our analysis and to the fact that we forced reconstruction using only one motor module, the changes reported in the weighting coefficients may reflect either a modification in the actual shape of the synergy itself or the override of a concurrent direct cortical command. It needs to be pointed out that, from our analysis, we cannot exclude that the changes that we observe in the synergy shape may depend from the differential recruitment of two (or more) independent muscle synergies.

However, results from previous studies reporting on the robustness of muscle synergies in different situations, including fatigue [56, 57], would suggest that the modifications that we observed are most likely due to an additional cortical drive overlapping the synergistic one. Also, the absence of changes in *R*^2^ between *baseline* and *task failure* for both exercise intensities further supports the possibility that the original synergistic structure is preserved and suggests that the overlapping command could be mediated by the same synergistic spinal structures.

### 4.2. Coherence Analysis

Together with the changes in the synergistic module, we showed a decrease in the overall cross-muscle coherence at *task failure*. We interpret the pooled coherence measure as proportional to the linear summation of two contributes: the synergistic command common to the three muscles and the components of the neural drive unique to each muscle that are synchronous among them. Under this interpretation, the decrease that we observed in pooled coherence could be due to either a decrease in synergistic activation or to a desynchronization of the unique neural drive to the muscles. Analysis of within-muscle coherence at task failure using motor unit spike trains decoded from the electromyographic signals has been reported to increase in muscles of both the upper and lower limbs [7, 8] due to an intensification of the cortical demand associated with maintaining task stability during fatigue. Also recently, Reyes and colleagues [58] have reported a diminished beta (15–30 Hz) band intermuscular and corticomuscular coherence contributions in two synergistic hand muscles during a spring compression task, suggesting a disengagement of the two muscles at the level of motor cortex when the force becomes highly unstable. These results are comparable with ours as also in our task there is an inherent instability induced progressively by the approaching of voluntary exhaustion. Hence, we can speculate that the decrease in pooled coherence is most likely due to a decrease in the synergistic command. The results we obtained from the pairwise and residual coherence analyses can help clarify this speculation.

The pairwise coherence can also be modelled as the summation of two terms: the one relative to the entire synergy (“*pairwise*” but with elements synchronized to the activity of the third muscle involved) and the one solely related to the pair of muscles considered (“*residual*,” excluding the effects of the third one). According to this assumption, the decline in pairwise coherence, consistent across both exercise intensities, could be explained either by a decrease in the coherence contribution relative to the synergistic drive (the command that is common to all three muscles) or by a desynchronization of the volley that is solely common to the two muscles.

**Figure 7:**
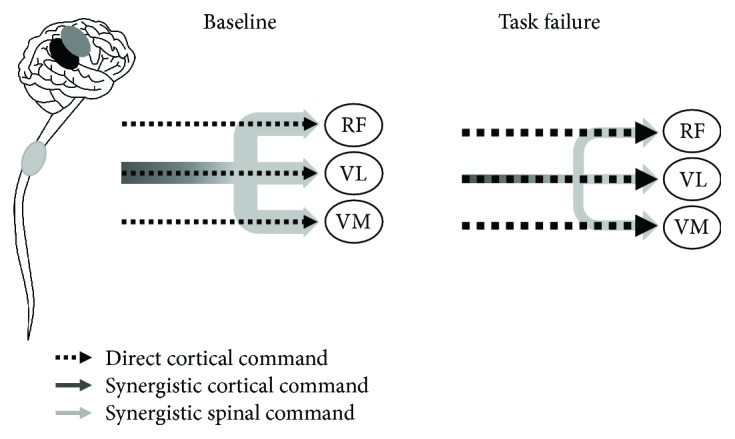
Conceptual model of the possible hypothesis suggested for the CNS to regulate the activity of synergistic muscles at task failure. At baseline, the three muscles receive a direct independent cortical command to each muscle (represented as the black pointed arrow) and a synergistic one of both cortical and spinal origin (represented as the common arrow that shades from dark grey, cortical component, to light grey, spinal component). When task failure occurs, the CNS suppresses the synergistic activation (represented as the common solid arrow becoming thinner) in favour of an increased cortical drive to the single muscle (represented as the individual pointed arrows becoming thicker) to keep the level of performance.

The fact that we did not observe changes in the residual coherences seems to suggest that task failure induces a decrease in contribution in the synergistic command. This speculation is further supported by the results reported in Figure 6. The percentage with which the residual coherence contributes to the total coherence increases for both LIE and HIE, indicating a diminished contribution of the synergy to the pairwise one. Combining these observations with those derived from the temporal analyses (namely the increase in muscular activation and the consistent modification of the synergy shape), we are encouraged to assume that task failure induces a decrease in synergistic drive and that the increased activity registered is likely to be due to an increase in direct cortical command to the muscles (Figure 7).

Nevertheless, we cannot exclude that the changes observed in intermuscular coherence could also be due to modifications in the common reflex inputs that may be independent from the descending drive. Changes in muscle spindles firing and an enhancement in presynaptic inhibition of Ia afferents have been observed during sustained contractions [59]. In fact, the increase in presynaptic inhibition could decrease the contribution of the common reflex drive to the motoneuronal pools. Following this interpretation, our results would suggest an overlap in the reflex projections across all the three analyzed muscles.

### 4.3. Differences between Exercise Intensities

Yet, in our study, most of the changes showed are found to be significant only for the higher intensity exercise (HIE). Many factors may account for this different behaviour. First, time to task failure was significantly different between LIE and HIE, and, with it, the different strategies adopted by the CNS to counterbalance exhaustion. Some studies have previously reported different ways the CNS approaches task failure at different force levels [7, 59]. Repetitive maximal or almost maximal force contractions lead to a full recruitment of motor units and a reduced facilitation of the Ia fibres together with the gain of the muscle spindles [59]. On the contrary, at low force intensities, the CNS tries to compensate for the decline in performance: by recruiting and rotating the motor units involved in the task, by modulating their discharge rate [59], and/or by intensifying the cortical drive [7, 16]. In our case, we can suppose that at HIE all three muscles of the quadriceps actively participating in the knee extension have reached almost the full recruitment of muscle fibres [60]. Therefore, there is less variability in the data at HIE when compared to LIE, due to the ensemble of all the physiological mechanisms regulated and imposed by the CNS to counteract the task failure and exhaustion.

### 4.4. Comparison with Similar Studies in the Literature

A few other studies have investigated intermuscular coherence through the surface EMG signal in synergistic muscles in normal [58, 61] and pathological conditions [62] and also in the presence of task failure [29, 63]. Most of these studies have observed coherence contribution in the beta band, with an increase related to task failure. In our study, we performed a different preprocessing of the EMG data [25]. Specifically, we chose not to rectify the EMG signal prior to coherence calculation. In fact, rectification process leads to a compression of the EMG spectrum towards lower frequencies [64], otherwise not possible due to the bandwidth limit of the EMG signal. In virtue of this choice, the EMG frequency spectra that we observed have only minimal contributions in the beta band and it is then not possible for us to see coherence contributions within that band. Therefore, our results cannot be compared to those previously reported by others upon intermuscular coherence, which use EMG rectification before performing coherence analysis.

## 5. Conclusions

In this study, we observed that task failure is associated with a modification of the synergistic recruitment of the quadriceps muscles during dynamic leg extension tasks which conveys a diminished overall synchronicity in the neural drive to the three muscles. Our results indicate that task failure does not alter the modular structure of muscular activation but is rather characterized by an increase in nonsynergistic command to the muscles that is employed to maintain the level of performance in the face of the decrease in mechanical efficiency. Our results further confirm the solidity of the muscle synergy hypothesis and the use of intermuscular coherence measures applied to standard surface EMG recordings to estimate the neural drive to the muscles.

## Figures and Tables

**Table 1:** EMG-RMS values (mV) for all muscles (RF, VL, and VM) and for both exercise conditions (LIE and HIE). Results are presented as the mean ± std. Significance is shown for comparison baseline versus task failure. ^∗∗^*P* < 0.01.

Muscles	LIE			HIE		
	Baseline	Task failure		Baseline	Task failure	
RF	62.1 ± 21.6	110.1 ± 54.6	^∗∗^	112.4 ± 48.7	174.7 ± 80.1	^∗∗^
VL	98.2 ± 48.2	143.4 ± 94.0	^∗∗^	146.6 ± 65.9	196.2 ± 98.4	^∗∗^
VM	65.2 ± 41.6	101.0 ± 72.4	^∗∗^	124.1 ± 74.6	164.6 ± 79.3	^∗∗^

## Data Availability

The data used to support the findings of this study are available from the corresponding author upon request.
